# Morbidities and mortality among hospitalized patients with hypopituitarism: Prevalence, causes and management

**DOI:** 10.1007/s11154-024-09888-8

**Published:** 2024-05-27

**Authors:** Fahim Ebrahimi, Lukas Andereggen, Emanuel R. Christ

**Affiliations:** 1 Department of Endocrinology, Diabetes and Metabolism, University Hospital Basel, Basel, Switzerland; 2 Department of Medical Epidemiology and Biostatistics, Karolinska Institutet, 17177 Stockholm, Sweden; 3 Department of Gastroenterology and Hepatology, Clarunis University Center for Gastrointestinal and Liver Diseases, Basel, Switzerland; 4 Department of Neurosurgery, Cantonal Hospital Aarau, Aarau, Switzerland; 5 Faculty of Medicine, University of Bern, Bern, Switzerland

**Keywords:** AVP deficiency and resistance, Hormone replacement therapy, Hypopituitarism, Metabolic syndorme, Morbidity, Mortality, Outcomes, Treatment

## Abstract

Hypopituitarism is a highly heterogeneous multisystem disorder that can have a major impact on long-term morbidity and mortality, but even more so during acute medical conditions requiring hospitalization. Recent studies suggest a significant in-hospital burden with prolonged length of stay, increased rate of intensive care unit (ICU) admission, and initiation of mechanical ventilation − all of which may lead to an increased risk of in-hospital mortality. On the one hand, patients with hypopituitarism are often burdened by metabolic complications, including obesity, hypertension, dyslipidemia, and hyperglycemia, which alone, or in combination, are known to significantly alter relevant physiological mechanisms, including metabolism, innate and adaptive immune responses, coagulation, and wound healing, thereby contributing to adverse in-hospital outcomes. On the other hand, depending on the extent and the number of pituitary hormone deficiencies, early recognition of hormone deficiencies and appropriate management and replacement strategy within a well-organized multidisciplinary team are even stronger determinants of short-term outcomes during acute hospitalization in this vulnerable patient population. This review aims to provide an up-to-date summary of recent advances in pathophysiologic understanding, clinical implications, and recommendations for optimized multidisciplinary management of hospitalized patients with hypopituitarism.

## Hypopituitarism and risk of hospitalization

In physiology, the anterior pituitary can be considered as the master gland of hormones with the main function of providing sufficient energy for a given clinical situation by regulating metabolism mainly via stimulation of peripheral endocrine glands, such as thyroid (TSH), adrenal (ACTH) and organs such as the liver (GH—IGF-1) [1]. In addition to the important control of metabolism, the regulation of reproduction (FSH, LH) and lactation (prolactin) are important functions of the anterior pituitary. However, while energy availability is critical in hospitalized patients with hypopituitarism, reproduction and lactation become less of a priority. This is also evidenced by the fact that secondary gonadal insufficiency can occur with low energy intake (e.g., excessive exercise or anorexia) as a functional adaptation of the organism to inadequate energy intake [2].

The posterior pituitary is involved in water homeostasis (ADH), vasoconstriction during volume depletion (ADH), and induction of labor during birth [3]. While water homeostasis and the ability to maintain blood pressure via vasoconstriction are important effects to maintain physiology in situations of acute illness [3], induction of labor however, becomes less important in such circumstances.

Hypopituitarism is a rare and heterogeneous condition with a multitude of factors characterizing the burden of the disease: (i) the underlying condition leading to the dysfunction of the gland (secreting or non-secreting pituitary tumors or non-tumoral causes), (ii) the extent and number of deficient anterior pituitary hormone axes (partial vs. complete deficiencies), (iii) affection of the posterior gland leading to arginine vasopressine deficiency (AVP; i.e., diabetes insipidus; DI), (iv) duration of inadequate or non-physiological replacement therapy, (v) post-treatment complications of surgery or radiation, and (vi) early detection and treatment of metabolic complications.

Most commonly, patients with known hypopituitarism are hospitalized for medical or surgical conditions that are unrelated to the underlying pituitary disease. Much less frequently, hypopituitarism is the cause of the hospitalization and may even be initially unrecognized in a hospitalized patient. Because hypopituitarism is a rare condition, new-onset diagnosis during acute hospitalization is often not straightforward and physicians on the emergency department should be aware of the clinical signs of hypopituitarism, as well as the approach when caring for such individuals.

Depending on the extent of hypopituitarism, individuals may be at higher risk of hospitalization, especially when secondary AI [4] or AVP-deficiency [5] are diagnosed. In situations of higher physiological stress, such as infections [9] or acute trauma, relative under-supply can lead to adrenal crisis which is a life-threatening condition. Overall, patients with secondary AI suffer from more frequent and severe infections [7] due to an impairment of both the innate and adaptive immunity [8] and recent evidence suggests that during the COVID-19 pandemic, patients with secondary AI were at threefold (95% CI 2.16–3.98) higher relative risk of COVID-19 infection and more than 20-fold increased relative risk of hospitalization, when compared with reference individuals from the general population [6]. To the same extent, AVP deficiency resulting in uncontrolled diuresis can lead to life-threatening dehydration and hyponatremia and thereby increase hypovolemia and thereby increase in-hospital morbidity and mortality.

## Mortality of hospitalized patients with hypopituitarism

Early epidemiologic studies suggested that compared to the general population, individuals with hypopituitarism − in particular female patients – have an increased long-term standardized mortality ratio (SMR) due to a higher incidence of cardiovascular and cerebrovascular events [10, 11]. More recently, it has been suggested that the higher overall mortality might be also caused by unplanned hospitalizations and associated in-hospital morbidity and mortality.

When estimating the mortality risk in patients with hypopituitarism, it is important to keep in mind that hypopituitarism is a highly heterogeneous disorder, and therefore several confounding factors must be taken into account, such as associated cardiometabolic comorbidities, the number and degree of pituitary axis deficiencies, under- or over-supplementation of hormones, the underlying pituitary pathology causing hypopituitarism, and the quality of medical management during hospitalization. In the following, we will discuss each of these aspects and the current evidence regarding their contribution to the mortality risk in these patients (Table 1).

**Table 1:** Underlying causes of increased mortality in hypopituitarism

Causes of Increased Mortality in Hypopituitarism
**Demographics**
Female gender
Younger age at diagnosis
**Hormone deficiencies**
AVP-deficiency
Hypogonadism
**Replacement therapy**
Unphysiological replacement therapy (thyroxine or hydrocortisone)
Lack of hormone replacement therapy (GH)
**Underlying diagnosis of hypopituitarism**
Craniopharyngioma
**Therapeutic modalities**
Radiation therapy
Transcranial surgery

Summary of all factors that have been associated with a higher mortality rate in patients with hypopituitarism.

Individuals with hypopituitarism often present with clinical signs and symptoms of the metabolic syndrome including visceral adiposity, insulin resistance, dyslipidemia, hypertension, as well as metabolic dysfunction-associated steatotic liver disease [12]. As a result, it has repeatedly been shown that they have a higher prevalence of cardio- and cerebrovascular diseases than the general population [13–15]. Though, it has been debated whether the higher risk of developing metabolic features can be explained by undetected or untreated hormone axis deficiencies or rather unphysiological over-supplementations of some hormone axes. The diagnostic workup of hypopituitarism is quite complex, and especially partial deficiencies of hormonal axes may go unrecognized for years, as the diagnosis in such cases is particularly difficult and requires experience with the clinical picture and careful dynamic endocrine testing. Growth hormone deficiency (GHD) is one of the most commonly overlooked deficiencies, and thus left untreated in adulthood, despite being one of the first hormonal axes to become deficient in patients with pituitary disorders [16]. Several studies suggest that untreated GHD may be a major contributor of an increased cardiovascular risk profile [17–19]. In fact, several changes in body composition and metabolic profiles compatible with the metabolic syndrome [20] have been described due to untreated GH deficiency, including changes in body composition with increased abdominal fat mass [21], insulin resistance [22], an abnormal lipid profile [23], a prothrombotic profile [24] and a chronic state of low-grade inflammation [25]. In addition to the metabolic consequences, the progressive loss of muscle mass and the development of sarcopenia are relevant complications of long-term GHD that affect the risk of mortality in these patients [26]. In turn, all these detrimental changes associated with GHD can be effectively reversed by GH replacement therapy, which has been demonstrated in several previous studies [27–30]. However, no longitudinal study has so far been able to answer the question whether GH replacement therapy may beneficially affect the mortality risk in adults with hypopituitarism and GHD.

Another relevant factor, additionally contributing to the increased incidence of metabolic diseases in patients with hypopituitarism is a prolonged over-replacement with hydrocortisone in those individuals with a diagnosis of secondary AI [31]. Indeed, over-replacement is unfortunately quite common [32] and can lead to a significant pro-inflammatory state with weakened immune defense [33], which in turn increases the risk of infection and recurrent hospitalizations [4]. While GHD is usually not substituted during hospitalization for acute medical conditions, as previous evidence suggests an increased mortality associated with substitution during acute medical conditions [34], inadequate replacement therapy of secondary AI is a leading cause of in-hospital morbidity and mortality among patients with hypopituitarism and its adequate acute management still remains a major challenge [35]. A recent population-based study of hospitalized patients with secondary AI and propensity-matched controls from the general population showed that secondary AI was associated with significantly higher rates of ICU admission and intubation as well as a prolonged length-of-hospital stay by approximately 3.3 days (95% CI, 2.82 − 3.71) [36]. Furthermore, patients with secondary AI had an approximately 45% increased relative risk of hospital readmission even up to one year after the index hospitalization, highlighting the high burden of disease associated with secondary AI. In contrast, in-hospital mortality rates were not increased when compared to matched controls, so the higher ICU admission rates could even be interpreted as an increased vigilance for the burden of secondary AI and a heightened diligence in the care of such patients [36].

Another hormonal axis that usually poses a unique challenge to clinicians caring for patients with hypopituitarism is the involvement of the posterior pituitary gland and the development of overt AVP deficiency (i.e., diabetes insipidus) [37]. Maintaining fluid balance during critical illness in these patients is often very challenging and time-consuming, yet states of under- or over-correction are common [38]. Hypernatremia, a common complication in these patients, has been associated with increased mortality in the ICU [39–41]. In fact, a large population-based, propensity score-matched cohort study of patients with hypopituitarism hospitalized for acute medical conditions recently demonstrated that hypopituitarism was independently associated with increased in-hospital mortality with an odds ratio (OR) of 1.32 (95% CI, 1.06 − 1.65) [42]. The increased mortality was primarily seen in individuals with concomitant AVP deficiency (OR 3.27; 95% CI, 2.22 − 4.83), whereas it was not increased in individuals with hypopituitarism without posterior pituitary deficiency (OR 0.78; 95% CI, 0.60 − 1.03) [42]. However, questions remain as to whether the increased mortality observed in patients with AVP deficiency is due to impaired water homeostasis and a higher risk of hypernatremia or hypovolemia, or whether it reflects more extensive pituitary-hypothalamic disease.

The underlying cause of hypopituitarism also has a relevant impact on the mortality risk. Hypopituitarism associated with autonomous growth hormone secretion in acromegaly and autonomous ACTH secretion in Cushing's disease have both been associated with an increased mortality due to the metabolic consequences of the often-prolonged hormonal excesses [43, 44]. While these associations have been confirmed in several older cohort studies with two- to fourfold increased SMRs in Cushing’s disease [44] and by 1.3- to twofold increased SMRs acromegalic patients [45], recent data from Sweden indicate that with improved treatment alternatives and, thus a higher proportion of patients achieving biochemical control nowadays, a normal life expectancy can be expected especially in patients with acromegaly [46]. Furthermore, it has been elegantly demonstrated that those individuals who do not achieve sustained biochemical control still have higher mortality rates [45].

## Approach to hospitalized patients with hypopituitarism

The care of patients with hypopituitarism requires an orchestrated multidisciplinary approach, not only in the long-term outpatient setting, but even more so in the acute inpatient setting where any acute condition can become life-threatening without timely and appropriate management.

### Known hypopituitarism and hospitalization for an acute medical or surgical condition

**Fig. 1:**
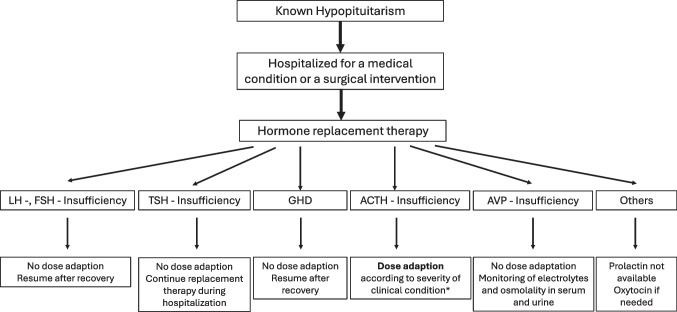
Hormone replacement therapy for hypopituitary patients hospitalized for a medical condition or a surgical intervention. *The corresponding dose adaptation is dependent on the severity of the underlying illness [47]

Pituitary hormone replacement therapy is usually performed by administration of the corresponding peripheral gland hormone (thyroxine, hydrocortisone), as there are no orally available drugs for ACTH or TSH [47, 48]. Sex steroids are replaced by depot injections, orally, or transdermally while GH replacement therapy requires daily (or soon weekly) subcutaneous injections [47, 49] (Fig. 1).

#### LH/FSH Insufficiency and sex hormone replacement therapy

Based on the above physiological considerations, gonadal axis replacement therapy is not critical in hospitalized patients with hypopituitarism. It can usually be resumed on an outpatient basis once the patient has recovered [47].

#### TSH Deficiency and thyroxine replacement therapy

 Although the effects of thyroid hormones are important for energy generation and maintenance of life-sustaining functions, a well-controlled replacement dose of thyroid hormone should usually not be changed in the event of acute hospitalizations [47]. This is mainly due to the long half-life of thyroid hormones not necessitating acute adjustment [47]. In addition, biochemical monitoring of thyroid function in acute medical conditions is usually difficult because TSH measurement cannot be used to guide the adequacy of thyroid replacement therapy [47, 48] and the well-established euthyroid thick syndrome further complicates the situation [50]. Therefore, we recommend that replacement therapy for TSH deficiency not be changed and that the adequacy of replacement therapy be reassessed in the outpatient setting. The only caveat is the assessment of adequate thyroid hormone absorption in critical illness, as gastrointestinal digestion and absorption may be impaired in specific conditions such as surgery of the gastrointestinal tract.

#### GHD and GH Replacement therapy

The metabolic effects of GH are well established: it induces lipolysis, promotes protein anabolism, and induces insulin resistance [51]. It may therefore attenuate the catabolic response in critical illness by shifting energy expenditure from carbohydrates to energy-dense lipids, thereby preserving proteins, and consequently muscle mass [51]. Although these effects sound attractive in the setting of critical illness, a randomized controlled trial involving more than 500 patients in Finland and in European countries in a mixed patient cohort (cardiac and abdominal surgery, acute trauma, or respiratory failure) requiring intensive care therapy for at least 5–7 days has shown that GH administration in such patients resulted in up to a twofold increase in mortality compared with placebo [34]. The underlying mechanisms leading to such findings have yet not been elucidated, but may be related to GH-induced insulin resistance with a tendency to higher blood glucose concentrations [52]. Indeed, higher glucose levels have been shown to negatively affect outcomes of patients in the ICU setting [52]. However, importantly in this study GH treatment was not given to hypopituitary patients with diagnosis of GHD. In addition, the dose administered was rather high [34]. Therefore, the results cannot be extrapolated to hypopituitary patients with GHD, but it may explain why GH replacement therapy is usually not administered to hospitalized patients with a medical or surgical condition, and replacement therapy is rather resumed after recovery. However, to our knowledge, there are no randomized controlled prospective data on GH replacement therapy in hospitalized patients with known hypopituitarism and GHD.

#### ACTH-Insufficiency and hydrocortisone replacement therapy

It is well established that adrenal crisis—defined as an episode of acute adrenal insufficiency (primary or secondary)—is a life-threatening situation requiring immediate action [47, 53]. Surprisingly, prospective studies evaluating the management of adrenal crisis are scarce, and the management of ACTH insufficiency is mainly based on exercise physiological studies and clinical experience, which may vary from place to place [47]. However, the basis for the administered dose is based on the dose usually administered in the outpatient setting and is increased according to the severity of the disease. Because illnesses requiring hospitalization—such as pneumonia or upper urinary tract infection without the need for intensive care—can be considered moderately severe, the administered hydrocortisone dose should be at least three to four times the usual daily dose, which should be administered in 3–4 daily doses [47, 54]. However, in- critical illness such as major trauma or sepsis requiring intensive care hydrocortisone, 100 mg should be administered as a bolus intravenously followed by 50 mg every 6 h [55]. Alternatively, a continuous infusion with hydrocortisone (200 mg/24 h) can be administered [55]. The dose of hydrocortisone is tapered with improvement of the clinical status (i.e., hydrocortisone 150 mg/24 h, then 100 mg/24). With further improvement oral administration of hydrocortisone is usually possible (double daily doses finally followed by the usual daily dose). or major surgical procedures such as heart valve replacement, bypass surgery, or Whipple's procedure, cortisol administration is similar to the situation of a hypopituitary patient requiring intensive care unit therapy, where the first IV bolus of hydrocortisone is administered with induction of anesthesia [47, 56]. For moderate surgical stress such as cholecystectomy or hernia repair, the administered dose of hydrocortisone may be reduced accordingly (i.e., 50 mg hydrocortisone initially followed by 100 mg/24 h) [56]. Of course, in case of postoperative complications (bleeding, infection), the tapering of hydrocortisone depends on the severity of the complications and the clinical course [56].

Importantly, ACTH insufficiency may be associated with significant hypovolemia that needs to be corrected. However, because the mineralocorticoid axis is functional in hypopituitary patients, electrolyte disturbances (hyperkaliemia-hyponatremia) are less common in ACTH-insufficiency than in primary AI [54]. Although prednisone or even dexamethasone can be administered, we prefer hydrocortisone because of its physiologic mineralocorticoid effect, which prednisone or dexamethasone do not have (dexamethasone) or to a lesser extent (prednisone) [47, 54].

Monitoring of the adequacy of hydrocortisone doses in hospitalized patients with ACTH insufficiency is mainly clinical [47]. Since overtreatment is more likely to be a consequence of chronic hydrocortisone overdose in the outpatient setting, the focus in hospitalized patients with ACTH insufficiency should be on avoiding undertreatment. Signs and symptoms of undertreatment include hypotension, weakness, and/or gastrointestinal symptoms.

#### AVP Insufficiency and ADH replacement therapy

 Patients with AVP insufficiency require adequate replacement during hospitalization. Because hospitalized patients (surgical and medical conditions) have impaired intravascular and extravascular volume status—most commonly hypovolemia—AVP insufficiency and its replacement can be a significant challenge [38]. n the case of a well-established dose of ADH, we prefer to maintain this dose during hospitalization and add fluids intravenously as needed. A combined approach of changing AVP doses and fluid administration at the same time is difficult to manage and may lead to overreplacement.

In addition to careful and regular assessment of volume status, monitoring of electrolytes (sodium) and osmolality in serum and urine (sometimes 2–3 times daily) is helpful in guiding fluid administration and, if necessary, ADH dose adjustment [38].

The modality of ADH administration depends on the clinical condition of the hypopituitary patient. ADH can be administered intranasally, sublingually, subcutaneously, or intravenously.

## Unknown hypopituitarism and hospitalization for a medical or surgical condition

The clinical features of acute hypopituitarism are usually defined by signs and symptoms of ACTH insufficiency, including fatigue, weakness, and hypotension, sometimes associated with gastrointestinal symptoms [57, 58]. Biochemically low sodium concentrations may be present in addition to inadequately low cortisol levels (which are often not assessed). The low sodium levels are a consequence of increased ADH secretion (with free water retention) to restore intravascular volume and compensate for inadequate ACTH-cortisol secretion [57]. In addition, signs and symptoms of local complications of pituitary disease may be present, such as severe headache, visual field defects, and diplopia due to nerve palsy [59].

**Fig. 2:**
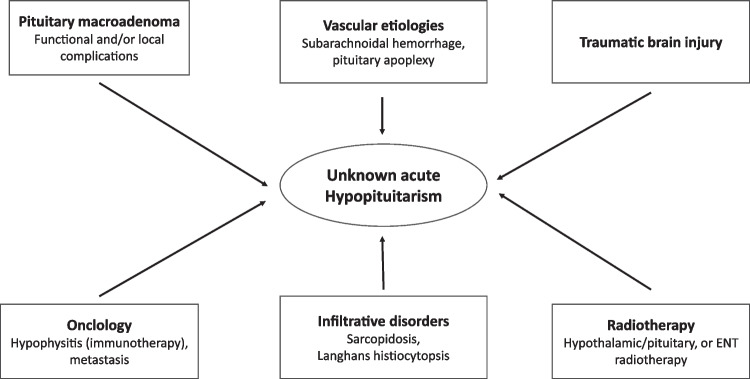
Possible mechanisms for the first manifestation of acute hypopituitarism in hospitalized patients without established diagnosis of hypopituitarism

The etiology of previously unknown hypopituitarism is heterogeneous (Fig. 2) and includes pituitary lesions, most commonly unknown pituitary adenomas that affect pituitary function [59]. A vascular etiology such as pituitary apoplexy is extremely rare, is most often associated with severe headache, and may be related to a difficult delivery with volume depletion [60] or to an unknown adenoma with acute bleeding [61]. In an oncologic context, hypopituitarism may occur when immunotherapy is administered, leading to hypophysitis with mainly ACTH insufficiency [62]. Metastases from other primary solid tumors are rare, but usually first manifest with ADH-insufficiency [63]. In the context of brain injury, at least partial hypopituitarism (gonadal axis) is frequently documented, but persistent hypopituitarism occurs only in max. 11% of these patients [64, 65]. Similarly, radiation therapy to head and neck structures or the pituitary gland can lead to hypopituitarism over time and should be suspected in the presence of a corresponding clinical picture [66, 67].

Finally, infiltrative diseases of the hypothalamic-pituitary region such as sarcoidosis or Langhans cell histiocytosis may cause hypopituitarism [68]. Similarly, metabolic disorders such as hemochromatosis with pituitary iron overload may result in hypopituitarism, at least in part [69].

Taken together, the signs and symptoms of ACTH deficiency with some clues from the personal history (oncologic disease, metabolic disease, known infiltrative/inflammatory disease, vascular disease, pregnancy, brain injury) and symptoms of local complications may help to establish the diagnosis of hypopituitarism, assess the appropriate biochemical parameters, and appropriately treat the patients with acute hypopituitarism.

## Management of hospitalized patients with hypopituitarism following neurosurgical procedures

This section focuses on perioperative management following both transcranial and transsphenoidal surgery. It provides an updated review of the clinical implications and recommendations for a well-coordinated, multidisciplinary approach to ensure optimal care of hospitalized patients with hypopituitarism following neurosurgical procedures.

Sellar masses often coincide with combined pituitary deficits, which may be caused by the tumor itself or by additional damage resulting from surgery, radiotherapy, or medical treatments [48]. It is hypothesized that the localization of somatotropic and gonadotropic cells, in conjunction with the vasculature of the pituitary system, may potentially contribute to the increased prevalence of GHD and LH-FSH insufficiency [70]. It has been shown that primary Gamma Knife radiosurgery for pituitary adenoma may result in lower rates of radiation-induced hypopituitarism compared to postoperative gamma knife radiosurgery, emphasizing the importance of minimizing doses to healthy pituitary tissue and ensuring ongoing endocrine follow-up [71]. Increasing the distance between the normal pituitary gland and the stereotactic radiosurgery target is associated with better preservation of anterior pituitary function, suggesting that maximizing this distance during adenoma resection is advisable [72].

For sellar and parasellar masses, surgical intervention via the transsphenoidal approach is commonly used [73, 74], particularly for craniopharyngiomas, where rates of hypothalamic obesity can be substantial in addition to long-term pituitary deficiency, a balanced approach between radical (including transcranial) resection and the need for effective disease control is recommended [75]. However, despite its minimally invasive nature, a transsphenoidal approach carries risks due to the intricate anatomy and surrounding neurovascular structures in a confined space [76]. As a result, hypopituitarism is not uncommon, with central AI being a significant concern that requires prompt recognition and treatment [77]. In addition, the occurrence of water metabolism disorders such as AVP insufficiency or syndrome of inappropriate antidiuretic hormone secretion (SIADH) is often transient, but requires repeated monitoring of serum and urine electrolytes and osmolality to prevent sudden fluctuations in blood sodium levels in hospitalized patients [78]. In clinical practice, sodium correction rates are often limited in patients with severe hyponatremia to prevent neurologic complications; however, this is associated with increased mortality and prolonged hospitalization [79] and pontine myelinolysis, although rare, can occur even with normal electrolyte levels [80]. Moreover, these risks can exacerbate pre-existing health problems in patients with hypopituitarism, disrupting physiologic balance and leading to longer hospital stays, increased intensive care unit admissions, readmission rates, and higher in-hospital mortality rates [81, 82]. In recent years, awareness and detection rates of hypopituitarism have increased not only in relation to the pituitary gland, but also in relation to other intracranial procedures, traumatic brain injury (TBI), and aneurysmal subarachnoid hemorrhage (aSAH), with studies indicating its presence in over 40% of perioperative cases. [In fact, increased rates of pituitary dysfunction following even moderate TBI and aSAH may increase both morbidity and mortality in affected individuals [83]. Potential mechanisms for hypopituitarism associated with TBI include direct injury to surrounding neurovascular structures and physical compression of anatomical structures, including the pituitary gland and stalk. SAH-induced hypopituitarism is related to the proximity of the circle of Willis to the hypothalamic-pituitary complex, which impairs pituitary function through direct compression, ischemia, increased intracranial pressure, and damage from neurosurgery, particularly affecting somatotrophic and gonadotrophic cells due to the vulnerable portal system as noted above [84–86]. Pituitary disorders are often overlooked in patients with TBI and aSAH because they present with non-specific symptoms, potentially leading to delayed recognition and a worse prognosis [87]. Specifically, clinical manifestations range from generalized fatigue and headache to more specific signs such as amenorrhea or lack of libido, with the most common deficiencies reported being growth hormone, ACTH, and gonadotropins, although the prevalence may vary with time since injury [65, 88, 89]. Thereby, the acute phase after TBI or aSAH is characterized by clinically significant abnormalities such as ACTH-cortisol deficiency and salt-water imbalance, which can be life-threatening and associated with increased morbidity and mortality [90]. In the chronic phase, gonadotropin deficiency and GHD contribute to morbidity, with GHD being the most common alteration in patients evaluated 6 months or more after the traumatic event [91–93]. These long-term effects can lead to cognitive impairment, metabolic problems, and decreased quality of life, often overlapping with symptoms of post-concussive syndrome and requiring careful differential diagnosis for appropriate management [93, 94]. Hormone replacement therapy is initiated upon diagnosis of hypopituitarism, particularly in cases of severe GHD [89]. Thereby, interdisciplinary expert recommendations underscore the importance of conducting endocrine assessments in SAH and TBI patients, emphasizing the timely screening for hypopituitarism post-injury or post-surgery [89]. Thus, understanding each of these conditions can enhance our grasp of the diverse nature of hypopituitarism.

In conclusion, these findings underscore the importance of early screening and identification of hypopituitarism in individuals undergoing intracranial procedures, which may have implications for treatment and rehabilitation strategies.

## Abbreviations

ACTH: Adrenocorticotropic hormone
ADH: Antidiuretic hormone
AI: Adrenal insufficiency
AVP: Arginine vasopressine
COVID-19: Coronavirus disease 2019
DI: Diabetes insipidus
GHD: Growth hormone deficiency
FSH: Follicle-stimulating hormone
HR: Hazard ratio
ICU: Intensive-care unit
IGF-1: Insulin-like growth factor-1
LH: Luteinizing hormone
OR: Odds ratio
PRL: Prolactin
aSAH: Aneurysmal subarachnoid hemorrhage
SIADH: Syndrome of inadequate ADH secrection
SMR: Standardized mortality ratio
TBI: Traumatic brain injury
TSH: Thyroid stimulating hormone
